# Scheduled Follow-Up Referrals and Simple Prevention Kits Including Counseling to Improve Post-Discharge Outcomes Among Children in Uganda: A Proof-of-Concept Study

**DOI:** 10.9745/GHSP-D-16-00069

**Published:** 2016-09-28

**Authors:** Matthew O Wiens, Elias Kumbakumba, Charles P Larson, Peter P Moschovis, Celestine Barigye, Jerome Kabakyenga, Andrew Ndamira, Lacey English, Niranjan Kissoon, Guohai Zhou, J Mark Ansermino

**Affiliations:** a University of British Columbia, Vancouver, Canada; b Massachusetts General Hospital, Boston, MA; c Mbarara University of Science and Technology, Mbarara, Uganda; d University of North Carolina at Chapel Hill, Chapel Hill, NC, USA

## Abstract

**Background:**

Recurrent illness following hospital discharge is a major contributor to childhood mortality in resource-poor countries. Yet post-discharge care is largely ignored by health care workers and policy makers due to a lack of resources to identify children with recurrent illness and a lack of cohesive systems to provide care. The purpose of this proof-of-concept study was to evaluate the effectiveness of a bundle of interventions at discharge to improve health outcomes during the vulnerable post-discharge period.

**Methods:**

The study was conducted between December 2014 and April 2015. Eligible children were between ages 6 months and 5 years who were admitted with a suspected or proven infectious disease to one of two hospitals in Mbarara, Uganda. A bundle of interventions was provided at the time of discharge. This bundle included post-discharge referrals for follow-up visits and a discharge kit. The post-discharge referral was to ensure follow-up with a nearby health care provider on days 2, 7, and 14 following discharge. The discharge kit included brief educational counseling along with simple preventive items as incentives (soap, a mosquito net, and oral rehydration salts) to reinforce the education. The primary study outcome was the number of post-discharge referral visits completed. Secondary study outcomes included satisfaction with the intervention, rates of readmission after 60 days, and post-discharge mortality rates. In addition, outcomes were compared with a historical control group, enrolled using the same inclusion criteria and outcome-ascertainment methods.

**Results:**

During the study, 216 children were admitted, of whom 14 died during hospitalization. Of the 202 children discharged, 85% completed at least 1 of the 3 follow-up referral visits, with 48% completing all 3 visits. Within 60 days after discharge, 22 children were readmitted at least once and 5 children (2.5%) died. Twelve (43%) readmissions occurred during a scheduled follow-up visit. Compared with prospectively enrolled historical controls, the post-discharge referral for follow-up increased the odds of readmission (odds ratio [OR], 1.92; 95% confidence interval [CI], 1.14 to 3.23) and care sought after discharge (OR, 14.61; 95% CI, 9.41 to 22.67). Overall satisfaction with the bundle of interventions was high, with most caregivers strongly agreeing that the discharge kit and post-discharge referrals improved their ability to care for their child.

**Conclusions:**

Interventions initiated at the time of discharge have the potential to profoundly affect the landscape of care during illness recovery and lead to significantly improved outcomes among children under 5 years of age.

## Abstract (precis)

Post-hospital discharge is a vulnerable time for recurrent illness and death among children. An intervention package consisting of (1) referrals for scheduled follow-up visits, (2) discharge counseling, and (3) simple prevention items such as soap and oral rehydration salts resulted in much higher health seeking and hospital readmissions compared with historical controls.

## BACKGROUND

In resource-poor countries, in-hospital death rates for children hospitalized for a serious infection are similar to death rates in the weeks after they return home.^1^ Health care workers, policy makers, and caregivers are often unaware of the high vulnerability during this post-discharge period and are poorly equipped to identify, triage, and provide definitive care for the children. An effective strategy is therefore required to address recurrent illness following hospital discharge in order to reduce overall childhood mortality.

This study builds on an earlier observational study in Uganda conducted between 2012 and 2014, in which we observed children who had been admitted to the hospital with proven or suspected infections, for the purpose of developing prediction models for post-discharge mortality.^2^ The predictive models included up to 5 variables, easily collected at admission, that identified children at high risk of mortality during the critical post-discharge period. These models can help direct resources to the most vulnerable children, but any interventions must be evaluated to determine their impact on morbidity and mortality before implementing at scale.

The objective of this proof-of-concept study was to determine the effectiveness of a discharge package—consisting of a discharge kit (including educational counseling and simple preventive items as incentives) and a post-discharge referral for scheduled follow-up visits—in improving families’ health-seeking behavior from a qualified provider, and ultimately in improving mortality during the post-discharge period.

## METHODS

### Design

This proof-of-concept study builds upon an earlier observational study in Uganda in 2012–2014, which had the primary objective to derive models to predict post-discharge mortality.^2^ The current study described in this article was conducted between December 2014 and April 2015 and represents the interventional continuation of the observational study. This study included an intervention aimed at improving outcomes following hospital discharge, whereas the earlier study was purely observational. The earlier study did not implement any systematic post-discharge policy. All children received routine care during enrollment and the post-discharge period. It is common practice to discharge children with instructions to return to a health center or hospital in the event of recurrence or worsening of illness.

This study builds upon an earlier observational study, in which modeling predicted post-discharge mortality among children. This proof-of concept study provided a post-discharge intervention to improve mortality.

### Population

This study was conducted at 2 sites—the Mbarara Regional Referral Hospital and the Holy Innocents Children’s Hospital, both in Mbarara, Uganda. The Mbarara Regional Referral Hospital is a public hospital funded by the Uganda Ministry of Health and is associated with the Mbarara University of Science and Technology Faculty of Medicine. The pediatric ward admits approximately 5,000 patients per year. Holy Innocents Children’s Hospital is a Catholic children’s hospital offering subsidized fee-for-service outpatient and inpatient care in Mbarara and admits approximately 2,500 patients annually. These study sites were chosen to reflect the relatively high proportion and use of both private and public hospitals in Uganda, and to improve external validity by comparing 2 types of institutions.

The earlier observational study was approved by the institutional review boards at the University of British Columbia (Vancouver, Canada) and the Mbarara University of Science and Technology (Mbarara, Uganda), the details of which have been published.^2^ This proof-of-concept study was separately reviewed and approved by the institutional review boards at the University of British Columbia (Vancouver, Canada) and the Mbarara University of Science and Technology (Mbarara, Uganda). The study was funded by Grand Challenges Canada.

### Eligibility

Using the same criteria as the observational study,^2^ children who were eligible to enroll in the proof-of-concept study were between the ages of 6 months and 5 years, and were admitted to the hospital with a proven or suspected infection. Subjects already enrolled in the study were not eligible to enroll again for subsequent admissions, nor were subjects residing outside of the official catchment area of the hospital (10 surrounding districts).

### Study Procedure

The proof-of-concept study used the same research nurses, field officers, and all equipment as in the earlier observational study. Following enrollment, a research nurse obtained and recorded clinical signs and symptoms as described in the observational study.^2^ The clinical care provided during the study was in accordance with local and national guidelines and reflected the in-hospital procedures used in the observational study (with the exception of the intervention itself). This was done to ensure a high degree of consistency when comparing the 2 cohorts.

### Interventions at Discharge

During enrollment in the study, children received routine care according to the Uganda National Guidelines until the point of discharge. At discharge, the children received a bundle of interventions. The bundle consisted of (1) post-discharge referral for follow-up visits organized by the research nurse at the time of discharge, and (2) a discharge kit consisting of counseling and simple preventive items to reinforce the counseling.

During the discharge counseling, the research nurse provided the child’s caregiver with a paper referral form for follow-up with either a community health worker or at a nearby health center on days 2, 7, and 14 following discharge. These days were chosen as the highest-risk times during the early post-discharge period.^1^^,^^2^ Before this study, we collected information on community health workers and health centers in the catchment area (10 districts) at the parish level. Caregivers could choose either a community health worker or a health center for the child’s follow-up visits, based on preference and proximity to their home. For caregivers who chose a follow-up visit at a health center, nurses provided a list of health centers (private and public) from which the caregivers could choose. The referral form was then addressed to either the health center or the community health worker, and caregivers received relevant information as written instructions.

The caregivers also received a discharge kit, which included brief educational counseling paired with simple preventive items as incentives to reinforce the education. The educational counseling consisted of a storyboard-style, laminated card written in the local language (supplementary material). Using this card, the nurse explained the child’s vulnerability during the discharge period and discussed 3 main themes of action: (1) prevention through hygiene and other health behaviors (e.g., mosquito net use), (2) recognition of signs of early illness recurrence, and (3) prompt care sought at a health center or from a community health worker. In our earlier research, these themes featured prominently in the children who died following discharge.^3^ Caregivers received the card along with 3 preventive items meant to reinforce the education (a mosquito net, 1 kg of soap, and 5 sachets of oral rehydration salts). The value of these household incentives was approximately US$6.50. The time it took hospital staff to give the bundle of interventions was less than 30 minutes.

At discharge, the nurse explained the child’s vulnerability during the discharge period and 3 main themes of action: prevention, recognition, and seeking care.

Approximately 60 days after discharge, field officers visited all subjects at their homes to determine vital status and assess whether care had been sought at a health center or from a community health worker. Field officers also administered a short survey to caregivers of the children to solicit feedback on the discharge intervention and the post-discharge referral for follow-up. Field officers were trained, prior to the earlier observational study, in patient tracking and administering questionnaires, and they had some health-related training. The procedures used to ascertain outcomes were the same in both the observational study and this study.

Study data were collected electronically using tablet computers and managed using the Research Electronic Data Capture (REDCap) tool hosted at the Child and Family Research Institute in Vancouver, Canada.^4^ REDCap is a secure, web-based application designed to support data capture for research studies.

### Analysis

#### Outcomes

The primary study outcome was the proportion of children who successfully completed at least 1 post-discharge referral for a follow-up visit at a health center or with a community health worker. Secondary study outcomes included caregiver satisfaction with the interventions (the discharge kit and post-discharge referral) and comparisons in post-discharge mortality, readmission, and care sought between this study and the earlier observational study.^2^

#### Statistical Analysis

We performed descriptive analyses of the primary outcome and of caregiver satisfaction. Using logistic regression, we also performed comparative analyses between the earlier observational study and this study. In this analysis, the outcomes of readmission and post-discharge health seeking were adjusted for both site of enrollment and the post-discharge mortality risk score. The post-discharge mortality risk score is a 5-item composite score including mid-upper arm circumference, oxygen saturation, time since most recent hospitalization, HIV status, and coma score. We also conducted a secondary univariate logistic regression analysis examining factors associated with completing post-discharge referrals. For the secondary analysis, a sample of 200 children would provide 80% power to detect a 10% absolute difference in health seeking compared with the historical cohort, at an alpha of .05. All analyses were conducted in SAS 9.4 (Cary, North Carolina, USA).

## RESULTS

A total of 216 children were enrolled in the study. An additional 93 children were screened but excluded from the study, mostly because they presented with a non-infectious illness (Figure). The median age was 16.1 months (IQR 10.2–29.1), and the sample was nearly evenly split between boys and girls (107 children, or 49.5%, were boys) (Table 1). Forty-two children (20%) were referred for the initial hospital admission, mostly from health centers. Fifty-three (24.7%), 59 (27.4%), and 56 (26.2%) children were underweight, stunted, or wasted (defined as height-for-age *z* score less than -2). One hundred and four (48%) were diagnosed with pneumonia, while 45 (20%) were diagnosed with malaria and 17 (8%) with diarrhea. The rate of in-hospital mortality was 6.5%.

**FIGURE:**
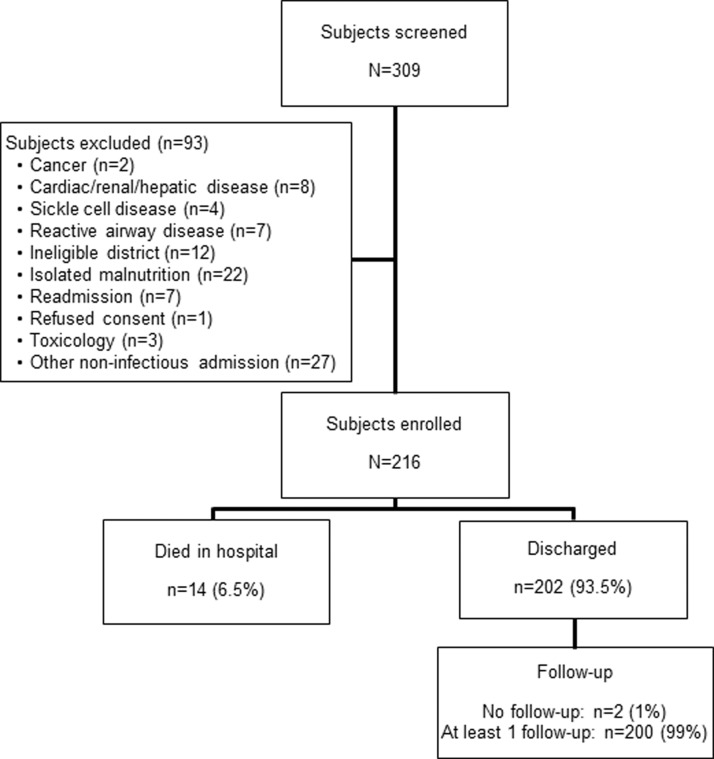
Study Flow of Subjects Enrolled and Excluded

**TABLE 1:** Baseline Characteristics of Study Subjects (N = 216)

Variable	Value
Male sex, No. (%)	107 (49.5)
Age, months, median (IQR)	16.1 (10.2, 29.1)
Prior care sought for illness, No. (%)	160 (74.1)
Referred for the initial hospital admission	42 (19.4)
Referral source: hospital	3 (7.0)
Referral source: health center	33 (78.6)
Referral source: untrained health worker	6 (14.3)
MUAC <115 mm, No. (%)	14 (6.5)
MUAC 115–125 mm, No. (%)	21 (9.7)
Underweight (WAZ <-2), No. (%)	53 (24.7)
Severely underweight (WAZ <-3), No. (%)	24 (11.2)
Wasted (WHZ <-2), No. (%)	56 (26.2)
Severely wasted (WHZ <-3), No. (%)	28 (13.1)
Stunted (HAZ <-2), No. (%)	59 (27.4)
Severely stunted (HAZ <-3), No. (%)	31 (14.4)
HIV positive, No. (%)	15 (7.0)
Maternal education, No. (%)	
No school	18 (8.3)
Less than primary 3	17 (7.9)
Primary 4 to primary 7	91 (42.1)
Secondary 1 to secondary 6	60 (27.8)
Post-secondary	30 (13.9)
Discharge diagnosis, No. (%)	
Malaria	43 (19.9)
Pneumonia	104 (48.2)
Diarrhea	17 (7.9)
Discharged against medical advice, No. (%)	17 (8.0)
In-hospital mortality, No. (%)	14 (6.5)
Referred to higher level of care, No. (%)	4 (1.9)

Abbreviations: HAZ, height-for-age *z* score; IQR, interquartile range; MUAC, mid-upper arm circumference; WAZ, weight-for-age *z* score; WHZ, weight-for-height *z* score.

### Post-Discharge Referrals

The number of children who survived to discharge was 202 (93.5%). Of these, 170 (84%) completed at least 1 scheduled post-discharge follow-up visit, 143 (71%) completed 2 follow-up visits, and 96 (48%) completed all 3 follow-up visits. Among those children who did not complete all 3 follow-up visits, the 3 most commonly reported reasons for non-completion were (1) health system factors, such as a closed health center; (2) family factors, such as lack of transportation or high cost; and (3) the child’s family did not consider the visit important (Table 2). For the first post-discharge follow-up visit, 115 (68%) were completed at a local health center and 56 (33%) were completed by a local community health worker. For the second and third visits, the same provider was used in 84% and 80% of cases, respectively. Among the total referral visits (N = 407), 12 (2.9%) resulted in admission, 9 (2.2%) resulted in referral to a higher level of care, and 127 (31.2%) resulted in an outpatient-based intervention. Further details of the breakdown of these outcomes according to first, second, and third visit are detailed in Table 2. Outpatient interventions were defined as disease-specific treatments. While general health advice was commonly provided, this was not considered an outpatient intervention. Outpatient interventions were accepted in 121 (95%) cases.

84% of children surviving to discharge attended at least 1 follow-up referral visit after discharge, but less than half completed all 3.

**TABLE 2:** Post-Discharge Referral Completions and Outcomes Among Discharged Children

	No. (%)
Referral program completions (N = 202)	
At least 1 visit	170 (84)
At least 2 visits	143 (71)
All 3 visits	96 (48)
Outcome for visit 1 (n = 171)	
No intervention	111 (65)
Outpatient-based intervention	54 (32)
Admission	2 (1)
Referral to higher level of care	4 (2)
Outcome for visit 2 (n = 141)	
No intervention	88 (62)
Outpatient-based treatment	42 (30)
Admission	8 (6)
Referral to higher level of care	3 (2)
Outcome for visit 3 (n = 95)	
No intervention	60 (63)
Outpatient-based treatment	31 (33)
Admission	2 (2)
Referral to higher level of care	2 (2)
Reasons for missed referral visits (n = 104)	
Child not sick/visit not considered important	22 (22)
Child away	12 (12)
Child admitted	7 (7)
Child died	3 (3)
Forgot to go	15 (15)
Visit not possible (health system factors^a^)	16 (16)
Visit not possible (family factors^b^)	23 (23)

a Examples of health system factors include closed health center and unavailable community health worker.

b Examples of family factors include cost barriers, no transportation available, and husband denied permission.

There were no statistically significant findings in the secondary analysis of predictors of follow-up, although trends toward increased referral compliance were noted among those children with increasing mid-upper arm circumference or weight-for-age *z* score, who had used a mosquito net consistently, and who had a length of stay of less than 5 days during the initial admission. Age, sex, household crowding, sibling deaths, and age of the child’s mother were not associated with follow-up success (Table 3).

**TABLE 3:** Analysis of Factors Associated With Post-Discharge Referral Completion (N = 202)

Variable	OR (95% CI)	*P* Value
Sex (female)	1.03 (0.48, 2.21)	.93
Age (for each month increase)	1.00 (0.97, 1.02)	.77
Referral at initial admission	0.85 (0.32, 2.60)	.74
MUAC (for each 1 mm increase)	1.01 (1.00, 1.04)	.10
WAZ (for each 1 SD increase)	1.20 (0.96, 1.50)	.11
HIV positive	2.52 (0.32, 19.96)	.38
Crowding (for each additional household member)	1.09 (0.90, 1.31)	.38
Sibling death	1.03 (0.39, 2.72)	.95
Maternal age (for each 1-year increase)	1.01 (0.94, 1.08)	.81
Mosquito net use (always vs. no/sometimes)	2.00 (0.90, 4.47)	.09
Length of stay > 5 days	0.53 (0.24, 1.19)	.12

Abbreviations: CI, confidence interval; MUAC, mid-upper arm circumference; OR, odds ratio; SD, standard deviation; WAZ, weight-for-age *z* score.

### Post-Discharge Readmission and Mortality

During the 60-day post-discharge period, a total of 22 (11%) children were readmitted at least once, for a total of 28 admissions (Table 4). Of those children who were readmitted, 12 were admitted through a scheduled referral visit, and the remaining 10 through self-referral. During the post-discharge period, 5 children died (2.5%); 4 died at home and 1 during a readmission. The child who died in the hospital was seen twice at referral follow-up visits. The 4 who died at home were not seen at follow-up visits or any occasion; no care was sought from any health care provider before death.

Within 60 days after discharge, 11% of children were readmitted.

**TABLE 4:** Post-Discharge Mortality and Readmission Details (N = 202)

	No. (%)
Mortality within 60 days post-discharge	5 (2.5)
Location of death	
Home	3 (60.0)
Home of a relative or friend	1 (20.0)
Hospital	1 (20.0)
60-day post-discharge readmission	22 (10.9)
Once	16 (72.7)
Twice	4 (18.2)
Three times	2 (9.1)
Source of readmission (n = 28)	
Self-referral	16 (57.1)
Scheduled post-discharge referral	12 (42.8)

### Satisfaction With the Interventions

Approximately 60 days after discharge, field officers administered a short survey with 5 questions for caregivers to solicit feedback on the bundle of interventions (Table 5). Overall, more than 75% of caregivers strongly agreed that the education provided at discharge improved their ability to care for their child during the post-discharge period; 65% of caregivers strongly agreed that the simple preventive items provided were helpful in caring for their child. Among those who completed at least 1 referral (n = 170), 62% found that the referrals were very helpful in caring for their child and 75% found that the referrals were neither difficult nor inconvenient. Overall, 75% were very satisfied with the interventions. When asked what else could have been done to help with their child, the responses varied. However, 3 responses occurred often, with 82 (41%) respondents stating they wished the program had provided transportation for the post-discharge referrals, 47 (23%) stating they wished the program provided either food in the hospital or following discharge, and 41 (20%) stating they wished the educational component was administered at the time of admission.

75% of caregivers were very satisfied with the interventions.

**TABLE 5:** Caregiver Satisfaction With Interventions^a^

	No. (%)
Did the education provided at discharge improve your ability to take care of your child? (n = 191)	
Yes, strongly	147 (76.9)
Yes, somewhat	44 (23.0)
No	2 (1.0)
Were the soap, oral rehydration salts, and mosquito net helpful in better caring for your child after discharge? (n = 189)	
Yes, strongly	123 (65.1)
Yes, somewhat	66 (34.9)
No	1 (0.5)
Did you feel that the referrals were helpful in caring for your child after discharge? (n = 170)	
Yes, very helpful	105 (61.7)
Yes, somewhat helpful	54 (31.8)
Not sure	5 (2.9)
No	6 (3.5)
Did you find the referrals difficult/inconvenient? (n = 170)	
Yes, very difficult/inconvenient	3 (1.8)
Yes, somewhat difficult/inconvenient	32 (18.8)
Not sure	7 (4.1)
No, not difficult/inconvenient	128 (75.2)
Overall satisfaction with discharge kit and post-discharge referral (n = 195)	
Very satisfied	72 (36.9)
Somewhat satisfied	117 (60.0)
Not satisfied	4 (2.1)

a Sample size for the satisfaction indicators are slightly different, reflecting that not all children discharged (such as most who were discharged against medical advice) received the counseling and incentives, and not all caregivers participated in the satisfaction survey.

### Comparing the Two Cohorts

In the earlier observational study, 1,307 children were enrolled using the same enrollment criteria as this study. Of these, 65 died during hospitalization (5%), resulting in 1,242 live discharges (Table 6). During the first 60 days of follow-up, 41 (3.3%) children died, 72 (5.8%) were readmitted, and 383 (30.8%) sought care with a community health worker, health center, or hospital. During the current study that included a bundle of interventions, the rates of mortality, readmission, and health seeking were 2.5%, 10.9%, and 87.6%, respectively. We found a non-significant 25% lower odds of death at 60 days (odds ratio [OR], 0.75; 95% confidence interval [CI], 0.29 to 1.92), a nearly twofold higher adjusted odds of readmission (OR, 1.97; 95% CI, 1.14 to 3.23), and a 14-fold higher adjusted odds of seeking post-discharge care (OR, 14.61; 95% CI, 9.41 to 22.67).

Compared with the earlier observational cohort study, we found a 25% lower odds of death at 60 days, a nearly 2-fold higher adjusted odds of readmission, and a 14-fold higher adjusted odds of seeking post-discharge care.

**TABLE 6:** Comparison of Outcomes Between Earlier Observational Cohort (N = 1,242) and Current Intervention Cohort (N = 202)

Outcome	Earlier Observational Cohort, No. (%)	Current Intervention Cohort, No. (%)	OR (95% CI)
Readmission	72 (5.8)	22 (10.9)	1.97^b^ (1.14, 3.23)
Any visit	383 (30.8)	177^a^ (87.6)	14.61^b^ (9.41, 22.67)
Death	41 (3.3)	5 (2.5)	0.75 (0.29, 1.92)

Abbreviation: CI, confidence interval; OR, odds ratio.

a Also includes non-referral visit; therefore, the number in this table is higher than the 170 indicated in Table 2.

b Adjusted for site of enrollment and post-discharge mortality risk score.

The absolute increase in care seeking post-discharge increased from approximately 30% in the earlier observational study (with no intervention) to nearly 90% in the current study (with the intervention to promote health-seeking behavior post-discharge). This indicates that less than 2 children would need to receive this intervention in order for 1 additional child to receive in-person follow-up care who otherwise would not have received follow-up care after discharge. The characteristics of these 2 samples were similar. However, the earlier observational cohort had a higher proportion of patients with severe malnutrition. The interventional cohort had a substantially higher proportion of pneumonia and lower proportion of malaria (and thus a lower mean oxygen saturation). The proportion of children with HIV and of those with an abnormal Blantyre Coma Scale score was slightly higher in the interventional cohort (Table 7). When applying our previously derived post-discharge mortality prediction model,^2^^,^^5^ the 2 cohorts had similar risk profiles for predicted 6-month mortality, suggesting that the differences in outcomes are unlikely to be related to differing risk profiles among the 2 cohorts of children. In both the earlier observational study cohort and the current study cohort with interventions, the modeled median risk of death at 6 months post-discharge was 5.1% (IQR in earlier observational study, 2.8 to 9.3; IQR in current study, 2.6 to 9.5).

**TABLE 7:** Characteristics of Discharged Subjects, Comparison Between Earlier Observational Cohort (N = 1,242) and Current Intervention Cohort (N = 202)

	Earlier Observational Cohort	Current Intervention Cohort
Male sex, No. (%)	682 (54.9)	103 (51.0)
Age, months, median (IQR)	18.1 (10.8, 34.6)	16.2 (10.0, 29.0)
MUAC <115 mm, No. (%)	96 (7.7)	12 (5.9)
MUAC 115–125 mm, No. (%)	87 (7.0)	19 (9.4)
Severely underweight (WAZ <-3), No. (%)	188 (15.1)	20 (10.0)
Severely wasted (WHZ <-3), No. (%)	232 (18.7)	24 (11.9)
Severely stunted (HAZ <-3), No. (%)	187 (15.0)	28 (13.9)
Mean SpO2 at admission	94.0 (90.0, 96.0)	91.0 (85.5, 97.0)
Percent with abnormal BCS score (<5)	133 (10.7)	31 (15.4)
HIV positive, No. (%)	58 (4.7)	14 (7.0)
Maternal education, No. (%)		
Less than primary 3	250 (20.1)	29 (14.4)
Primary 4 to primary 7	630 (50.7)	85 (42.1)
Secondary 1 to secondary 6	269 (21.6)	58 (28.7)
Post-secondary	93 (7.5)	30 (14.9)
Discharge diagnosis, No. (%)		
Malaria	418 (33.6)	39 (19.3)
Pneumonia	390 (31.4)	98 (48.5)
Diarrhea	96 (7.7)	17 (7.4)
Discharged against medical advice, No. (%)	120 (9.6)	17 (8.4)

Abbreviations: BCS, Blantyre Coma Scale; HAZ, height-for-age *z* score; IQR, interquartile range; MUAC, mid-upper arm circumference; WAZ, weight-for-age *z* score; WHZ, weight-for-height *z* score.

## DISCUSSION

This proof-of-concept study evaluated the effectiveness of using a discharge bundle, consisting of a discharge kit (education and preventive items as incentives) and post-discharge referrals for follow-up visits, in a hospital environment in southwestern Uganda to improve health-seeking behavior and post-discharge mortality. Results showed that this intervention was both feasible and effective. After administering this intervention to the caregivers of some 200 children, we found that 89% of children successfully achieved at least 1 post-discharge follow-up visit, with nearly 50% completing all 3 follow-up visits. The observed falling compliance in referral completion, however, and the fact that 40% of missed visits were due to circumstances that appeared to be beyond the control of the caregiver, suggest that 2 visits may be more practical in bringing this type of intervention to scale.

Two, instead of three, follow-up visits post-discharge may be more practical in bringing this type of intervention to scale.

Post-discharge care was community focused, with referrals being directed to community health workers and nearby health centers. Three-quarters of caregivers reported that these visits were neither inconvenient nor difficult. Most strongly agreed that the education and referral were important components of care for their child’s recovery. Compared with prospective observational data collected before this study, this package of interventions was associated with increased hospitalizations (representing increased health seeking), with over 40% of readmissions being directly linked to a referral visit. Overall health seeking from a community health worker or a health center post-discharge was only 30% in the previous observational study with no intervention compared with nearly 90% in the current study that included the package of interventions to improve health seeking. This reflects an important achievement given that modeling in the observational study predicted that more than 1 of every 30 children discharged is likely to die within the first 2 months and that health-seeking behavior is poor for the most common causes of childhood death.^2^^,^^6^

Post-discharge mortality is a neglected but important issue in the field of pediatric global health. Prior research has shown that, in some areas, mortality after discharge exceeds mortality in the hospital.^1^ Improved post-discharge mortality is an important contributor to overall childhood mortality; however, policy and practice have not recognized its importance, and a systematic approach to post-discharge care is lacking in most resource-poor countries. While resources such as the Integrated Management of Childhood Illness strategy and the Emergency Triage Assessment and Treatment guidelines provide standardized approaches to the acute phase of infectious illness, no resources or guidelines currently exist to provide recommendations during the vulnerable recovery phase.

Post-discharge mortality is a neglected but important issue in the field of pediatric global health.

Although post-discharge care should be an important component in the care of all discharged children, limited resources and an already strained health system are likely to be major barriers in its implementation. The current context among hospitals in Uganda and elsewhere is that the limited post-discharge care that does occur focuses primarily on the ongoing treatment of specific diseases such as HIV and tuberculosis. We are not aware of any hospitals in resource-poor settings that have adopted general discharge policies. In addition to a lack of robust policies for post-discharge care, the larger issue is the availability of resources, which is a major limitation to the scalability of effective post-discharge care. At the community level, the capacity to complete follow-up visits is sufficient and does appear to exist in most areas, but the hospital resources to assess children for risk and to implement the referral process are likely to be insufficient. We propose the establishment of discharge nurses to oversee this role, as has been suggested for improving post-stroke outcomes in sub-Saharan Africa.^7^ Further, engagement with other key stakeholders (e.g., ministries of health, hospital administrations, and community health worker training programs) is critical in the development of a scalable intervention.

We propose that discharge nurses should assess children for risk and implement a referral process to improve health outcomes after discharge.

The identification of the most vulnerable children presents an important strategy to improve the cost-effectiveness of post-discharge care, as limited resources can be used to target high-risk children. Our group recently derived a clinical prediction tool that uses 5 easily collected variables to identify such children.^2^ This tool has been incorporated into a mobile application to enable frontline health workers to rapidly identify vulnerable children before discharge.^5^ The administration of the discharge bundle evaluated in this study could be an inexpensive and effective strategy to improve care following discharge, as it is well recognized that caregivers could benefit from additional education surrounding illness recognition and health seeking.^6^^,^^8^

Prior research has shown that a significant proportion of child deaths occur outside of health facilities.^9^ Our study found that 4 out of 5 children who died after discharge were outside of the health system, reflecting similar findings from our work during the observational study. Critical to addressing post-discharge care, therefore, is improved community-level care during the vulnerable post-discharge period, which was an important focus of our work.

### Limitations

This proof-of-concept study is subject to several important limitations. First, the small sample size limits the ability of this study to make robust comparisons with the earlier observational study. Although comparisons of study outcomes such as health seeking and readmission provide some insight into the potential value of a post-discharge intervention, a primary study outcome based on mortality is preferable and will be a primary component of future research. The results of this study, however, serve an important role in guiding the design and sample size calculations for subsequent research, as does the feedback provided by the participants in the study. A further limitation of this study, and similar studies, is that the incorporation of post-discharge follow-up in a research context may not be easily replicated in a non-research context. The discharge education given to caregivers, highlighting the vulnerability of their children during the post-discharge period, likely played an important role in motivating them to sacrifice time and money to complete the follow-up referral visits. Follow-up interventions, therefore, must be strongly linked to education at the time of discharge. A final limitation of this study is the relatively narrow age range (6 months to 5 years) of children. The reason this age range was chosen was based on design considerations for the earlier observational study, which we used to develop the prediction model. Our research group is currently working toward the expansion of these prediction models to eventually include both younger (<6 months) and older (>5 years) children. Finally, because this intervention consisted of a bundle of interventions, it is impossible to identify which components were critical in the results that were observed. The simple preventive items that complemented the education (a mosquito net, soap, and oral rehydration salt) were relatively expensive (approximately US$6.50) and may impact scalability. However, it is unlikely that these particular incentives are required in order to observe these benefits. The mosquito net was the most expensive item in the bundle (about US$5.75); it could easily be replaced with a less expensive item that complements the education while still providing value to the caregiver, and could serve as a means to reinforce the instructions provided by the discharge nurse.

## CONCLUSION

In conclusion, we found that a simple bundle of interventions at discharge, including brief educational counseling and a post-discharge referral, can improve post-discharge care and outcomes among children discharged from the hospital. New research is currently being planned to establish the benefit of a bundle of interventions at discharge to improve post-discharge mortality and will explore bringing such interventions to scale in Uganda.

## Supplementary Material

supplementary material

## Notes

**Peer Reviewed**

**First Published Online:** 2016 Sep 14

## References

[1] Wiens M Pawluk S Kissoon N. Pediatric post-discharge mortality in resource poor countries: a systematic review. PLoS One. 2013;8(6):e66698. 10.1371/journal.pone.0066698. 23825556 PMC3692523

[2] Wiens MO Kumbakumba E Larson CP Ansermino JM4 Singer J Kissoon N Postdischarge mortality in children with acute infectious diseases: derivation of postdischarge mortality prediction models. BMJ Open. 2015;5(11):e009449. 10.1136/bmjopen-2015-009449. 26608641 PMC4663423

[3] Wiens MO Childhood mortality from acute infectious diseases in Uganda: studies in sepsis and post-discharge mortality [thesis]. Vancouver (Canada): University of British Columbia; 2015 Available from: http://hdl.handle.net/2429/53787

[4] Harris PA Taylor R Thielke R Payne J Gonzalez N Conde JG. Research electronic data capture (REDCap)—A metadata-driven methodology and workflow process for providing translational research informatics support. J Biomed Inform. 2009;42(2):377–381. 10.1016/j.jbi.2008.08.010. 18929686 PMC2700030

[5] English LL Dunsmuir D Kumbakumba E Ansermino JM Larson CP Lester R The PAediatric Risk Assessment (PARA) mobile app to reduce postdischarge child mortality: design, usability and feasibility for health care workers in Uganda. JMIR mHealth uHealth. 2016;4(1):e16. 10.2196/mhealth.5167. 26879041 PMC4771927

[6] Geldsetzer P Williams TC Kirolos A Mitchell S Ratcliffe LA Kohli-Lynch MK The recognition of and care seeking behaviour for childhood illness in developing countries: a systematic review. PLoS One. 2014;9(4):e93427. 10.1371/2Fjournal.pone.0093427. 24718483 PMC3981715

[7] Ovbiagele B. Tackling the growing diabetes burden in Sub-Saharan Africa: a framework for enhancing outcomes in stroke patients. J Neurol Sci. 2015;348(1-2):136–41. 10.1016/j.jns.2014.11.023. 25475149 PMC4298457

[8] Sandberg J Odberg Pettersson K Asp G Kabakyenga J Agardh A. Inadequate knowledge of neonatal danger signs among recently delivered women in southwestern rural Uganda: a community survey. PLoS One. 2014;9(5):e97253. 10.1371/2Fjournal.pone.0097253. 24824364 PMC4019554

[9] Nair H Simões EA Rudan I Gessner BD Azziz-Baumgartner E Zhang JSF Global and regional burden of hospital admissions for severe acute lower respiratory infections in young children in 2010: a systematic analysis. Lancet. 2013;381(9875):1380–90. 10.1016/S0140-6736(12)61901-1. 23369797 PMC3986472

