# Supplementary material for: Scheduled Follow-Up Referrals and Simple Prevention Kits Including Counseling to Improve Post-Discharge Outcomes Among Children in Uganda: A Proof-of-Concept Study
Source: Glob Health Sci Pract. 2016 Sep 28;4(3):422–34. doi: 10.9745/GHSP-D-16-00069 (PMC5042698; doi:10.9745/GHSP-D-16-00069)
Supplement: supplementary material [file GHSP-D-16-00069_index.html]

Supplement to Scheduled Follow-Up Referrals and Simple Prevention Kits Including Counseling to Improve Post-Discharge Outcomes Among Children in Uganda: A Proof-of-Concept Study | Global Health: Science and Practice

## GHSP-D-16-00069 Supplementary Material

Wiens et al. doi: 10.9745/GHSP-D-16-00069

- Supplementary Material - Wiens et al. doi: 10.9745/GHSP-D-16-00069
