# Supplementary material for: Scheduled Follow-Up Referrals and Simple Prevention Kits Including Counseling to Improve Post-Discharge Outcomes Among Children in Uganda: A Proof-of-Concept Study
Source: Glob Health Sci Pract. 2016 Sep 28;4(3):422–34. doi: 10.9745/GHSP-D-16-00069 (PMC5042698; doi:10.9745/GHSP-D-16-00069)
Supplement: supplementary material [file 16-00069-Supplementary-material.pdf]

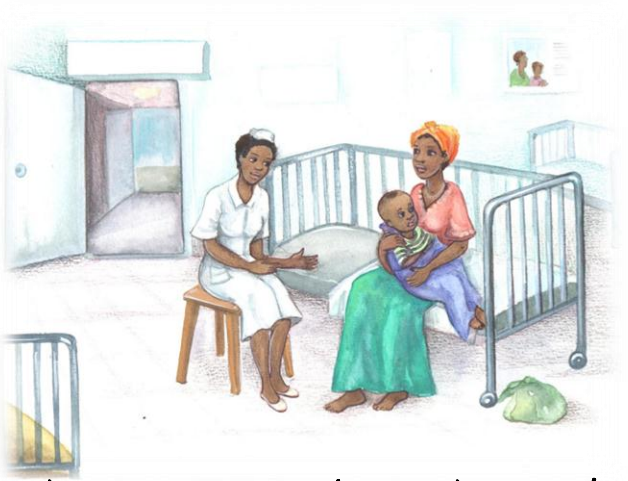

Don't forget - just because you have been discharged does not mean the illness if fully cured. Be vigilant! Practice good hygiene! Watch for danger signs! Seek care early and nearby!

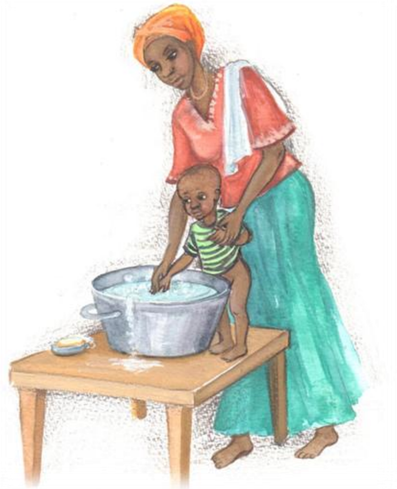

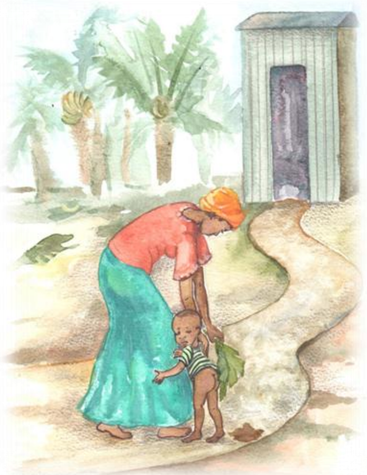

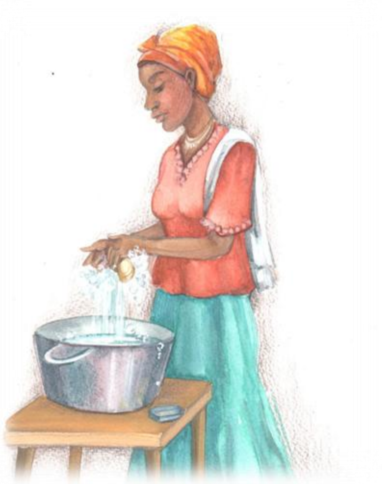

Practice good hygiene! Wash your hands often. Wash your child's hands often. Wash after using the latrine, cleaning your child and before eating.

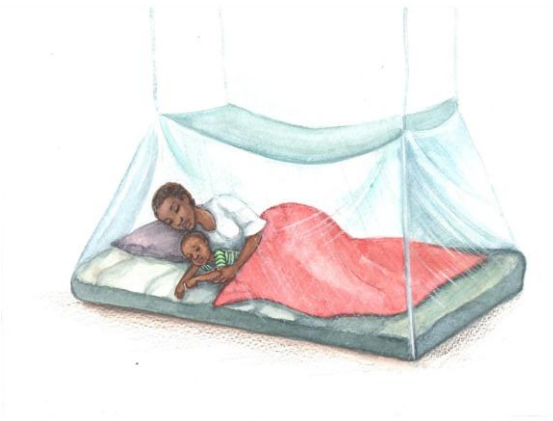

Always sleep under a mosquito net!

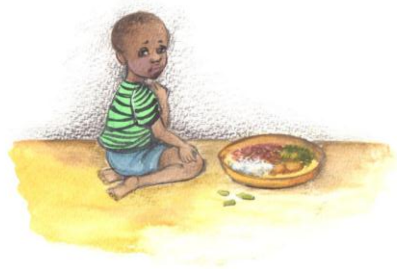 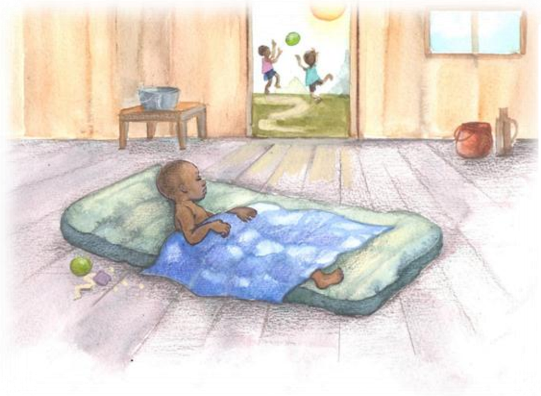 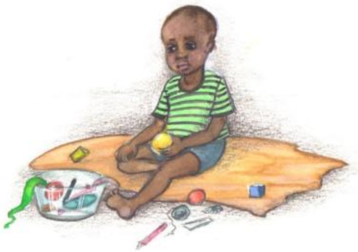

Watch for the danger signs! If your child is not eating, not playing or sleeping all day he might be very ill. Also watch for difficulty breathing, fevers and convulsions.

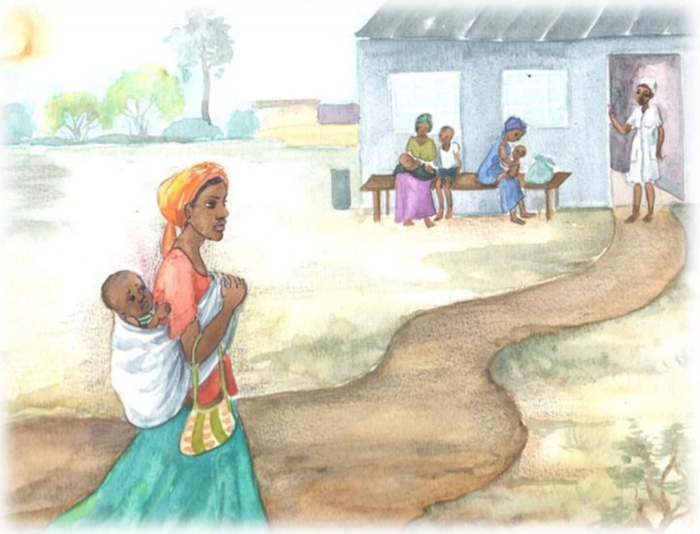

Seek care early and nearby! Do not delay seeking care. Go to the nearest health center or visit a member of your village health team.
